# Cardiovascular risk management beyond statins: review of new therapies available in Italy

**DOI:** 10.1186/s43044-025-00660-0

**Published:** 2025-07-01

**Authors:** Maurizio Capuozzo, Alessandro Ottaiano, Claudia Cinque, Stefania Farace, Francesco Ferrara

**Affiliations:** 1 Asl Napoli 3 SUD, Naples, Italy; 2 Istituto Nazionale Tumori di Napoli, IRCCS “G. Pascale”, Naples, Italy

**Keywords:** Statins, Bempedoic acid, Cholesterol, LDL-C, PCSK9 inhibitors, Dyslipidemia

## Abstract

**Background:**

Statins remain foundational in the prevention and treatment of atherosclerotic cardiovascular disease (ASCVD). However, despite optimal statin therapy, a significant residual risk of ASCVD persists, highlighting the need for novel lipid-lowering strategies targeting both low-density lipoprotein cholesterol (LDL-C) and other atherogenic lipoproteins. Over the last decade, a new generation of pharmacological agents has been developed to enhance dyslipidemia management beyond traditional statins.

**Main body:**

Bempedoic acid, a prodrug activated in the liver, inhibits ATP-citrate lyase (ACLY), an enzyme upstream of HMG-CoA reductase in the cholesterol synthesis pathway. This action reduces hepatic cholesterol synthesis and simultaneously upregulates LDL receptor expression, promoting enhanced LDL-C clearance. These dual actions provide a statin-independent approach to LDL-C reduction, particularly beneficial for patients with statin intolerance. In addition, proprotein convertase subtilisin/kexin type 9 (PCSK9) inhibitors, including monoclonal antibodies and siRNA-based therapies, have shown robust LDL-C-lowering effects by preventing LDL receptor degradation, leading to a significant reduction in cardiovascular risk. Other innovative lipid-modifying approaches include antisense oligonucleotides targeting apolipoprotein C3, angiopoietin-like protein 3, and lipoprotein(a) [Lp(a)]—such as pelacarsen and olpasiran—which demonstrate promising results in addressing genetically driven dyslipidemias. Additionally, strategies aimed at enhancing apolipoprotein A1 and promoting high-density lipoprotein functionality are under investigation, although clinical validation remains ongoing.

**Conclusion:**

This review underscores the evolving landscape of lipid-lowering therapies, with emphasis on agents acting through novel mechanisms beyond statin pathways. Bempedoic acid, by inhibiting ACLY and increasing LDL receptor expression, represents a safe and effective option for reducing LDL-C, especially in statin-intolerant individuals. PCSK9 inhibitors further expand therapeutic options by augmenting LDL receptor recycling and clearance. The integration of these agents into clinical practice may help mitigate residual cardiovascular risk and personalize treatment strategies in dyslipidemia management.

## Background

Atherosclerotic cardiovascular disease (ASCVD) remains the primary cause of death both in the USA and worldwide [1]. Characterized by the buildup of atherosclerotic plaques within the arterial walls, this condition can lead to severe cardiovascular events, including myocardial infarction and stroke [2]. Robust evidence from genetic, observational, and interventional studies has firmly established a dose-dependent relationship between low-density lipoprotein cholesterol (LDL-C) and the development of ASCVD [3]. In essence, higher levels of LDL-C are directly associated with an increased risk of ASCVD. Statins are among the most thoroughly investigated pharmacological agents and have demonstrated remarkable efficacy in lowering LDL-C levels. Owing to their proven effectiveness, they serve as the cornerstone of lipid-lowering therapy and are endorsed by major international guidelines as first-line agents for managing dyslipidemia and preventing cardiovascular events [4, 5]. Nevertheless, despite a substantial body of evidence confirming their efficacy and safety, statin intolerance—whether actual or perceived—is a frequent issue in clinical practice [6]. Commonly reported adverse effects include myalgia, fatigue, and gastrointestinal discomfort, often leading to treatment discontinuation or suboptimal adherence [7, 8]. This is particularly concerning given that poor adherence to statin therapy has been associated with a higher risk of major cardiovascular events [9]. Estimates of statin intolerance vary considerably, with a general prevalence near 10%. However, this rate differs notably between randomized controlled trials, which report intolerance in 6% to 8% of participants, and real-world longitudinal studies, where rates can climb to 30–35% [10]. These discrepancies likely arise from variations in study populations, methodologies, and definitions of intolerance. Complicating matters further is the nocebo effect, wherein patients develop adverse symptoms driven by negative expectations rather than pharmacological action. Notably, the SAMSON trial (NCT02668016) highlighted that a significant proportion of adverse reactions attributed to statins are actually nocebo responses [11, 76]. Despite such insights, maintaining adherence to statin therapy remains a significant clinical challenge [12]. Consequently, there is a growing need for alternative lipid-lowering strategies for patients who are unable or unwilling to take statins. Recently, several non-statin agents have emerged, offering new therapeutic avenues to lower LDL-C and mitigate ASCVD risk. Among these, bempedoic acid and PCSK9 inhibitors have shown particular promise, offering effective lipid-lowering options for patients with statin intolerance or insufficient response to statin therapy. These novel agents have the potential to substantially reduce cardiovascular risk and improve patient outcomes.

## Main text

### PCSK9

A key mechanism in cholesterol homeostasis involves the liver’s capacity to remove circulating low-density lipoprotein (LDL) particles through LDL receptor-mediated uptake on hepatocytes. Following internalization, LDL dissociates from its receptor within endosomes and is subsequently degraded, allowing the receptor to recycle back to the cell surface for further LDL clearance. This process is critically modulated by proprotein convertase subtilisin/kexin type 9 (PCSK9), which plays a pivotal role by binding to the LDL–LDL receptor complex. Upon this interaction, PCSK9 prevents the normal intracellular separation of LDL from its receptor, directing both components toward lysosomal degradation. Consequently, fewer LDL receptors are recycled to the plasma membrane, impairing the liver’s ability to remove LDL from the circulation [13]. Genetic studies have emphasized the essential regulatory function of PCSK9 in lipid metabolism. Gain-of-function mutations in PCSK9 are linked to elevated plasma LDL cholesterol levels and constitute a third genetic cause of familial hypercholesterolemia, alongside mutations affecting the LDL receptor and apolipoprotein B (apoB) [14]. In contrast, PCSK9 loss-of-function variants are associated with reduced LDL cholesterol concentrations [15, 16]. Remarkably, individuals who are homozygous for PCSK9 deficiency exhibit extremely low LDL cholesterol levels without apparent adverse health effects and retain normal reproductive capacity [17]. Mendelian randomization studies further support the causal relationship between PCSK9 function and cardiovascular risk, demonstrating that loss-of-function variants in PCSK9 are associated with lower levels of apoB and a reduced incidence of cardiovascular events [18, 19]. Additional data indicate that concurrent loss-of-function polymorphisms in both PCSK9 and 3-hydroxy-3-methylglutaryl-CoA (HMG-CoA) reductase confer synergistic protection against atherosclerotic disease [20, 21]. Collectively, these findings highlight the therapeutic value of incorporating PCSK9 inhibitors into statin-based regimens to achieve superior cardiovascular outcomes. Early efforts to develop PCSK9 inhibitors centered on the creation of monoclonal antibodies. Advances in antibody engineering made this therapeutic approach feasible, evolving from fully murine antibodies to chimeric, humanized, and ultimately fully human monoclonal antibodies. This progression significantly reduced immunogenicity, improving tolerability and minimizing the risk of neutralizing antibody formation [22]. The advent of anti-PCSK9 monoclonal antibodies has markedly expanded the therapeutic arsenal for LDL-C reduction, enabling many patients to achieve guideline-recommended targets [23, 24]. Randomized controlled trials have confirmed that anti-PCSK9 monoclonal antibodies not only lower LDL-C but also reduce the risk of ASCVD-related events. The landmark FOURIER trial (NCT01764633), which enrolled over 27,000 patients with established ASCVD receiving statins, demonstrated that treatment with evolocumab resulted in a nearly 60% LDL-C reduction from baseline and was associated with a 20% relative risk reduction in cardiovascular death, stroke, and myocardial infarction compared to placebo [25]. Similar benefits were observed in the ODYSSEY OUTCOMES trial (NCT01663402), involving more than 18,000 patients with a recent acute coronary syndrome on high-intensity statins. Participants randomized to alirocumab achieved LDL-C reductions of 62.7%, 61%, and 54.7% at 4, 12, and 48 months, respectively, and experienced a significant reduction in the composite endpoint of cardiovascular death, non-fatal myocardial infarction, non-fatal stroke, or hospitalization for unstable angina (9.5% vs. 11.1%). All-cause mortality was reduced by approximately 18%, and non-fatal cardiovascular events by 13% [26–28]. Importantly, both trials confirmed the favorable safety profile of PCSK9 inhibitors, with adverse event rates comparable to those in placebo groups. Beyond clinical outcomes, imaging studies have been instrumental in assessing treatment effects on atherosclerotic plaque burden. Serial intravascular ultrasound (IVUS) imaging has demonstrated that intensive LDL-C lowering, particularly with high-dose statins, can slow disease progression and even promote plaque regression [29]. In the ASTEROID trial (NCT00240318), high-intensity statin therapy with 40 mg daily of rosuvastatin reduced LDL-C to approximately 70 mg/dL while increasing HDL-C by 15%, resulting in significant regression of coronary atherosclerosis, as measured by all three prespecified IVUS endpoints [30]. Similarly, the PRECISE-IVUS trial (NCT01043380) demonstrated that combining statins with ezetimibe achieved superior reductions in plaque burden compared to statins alone, likely due to the additional LDL-C lowering via inhibition of cholesterol absorption [31]. The GLAGOV trial (NCT01813422) evaluated the impact of evolocumab (420 mg monthly) in patients with coronary artery disease already receiving statins. Evolocumab significantly reduced LDL-C and induced greater plaque regression, as assessed by IVUS, with a higher proportion of patients showing regression compared to placebo [32]. Further analysis using radiofrequency backscatter imaging revealed modest decreases in fibrous and fibrofatty components and a notable increase in calcification—findings aligned with prior observations from high-intensity statin therapy, suggesting that intensive lipid lowering facilitates both plaque regression and stabilization via calcification [33, 34]. The HUYGENS trial (NCT03570697) assessed monthly evolocumab (420 mg) versus placebo over one year in patients with non-ST-elevation myocardial infarction and high-risk plaque features. Utilizing both IVUS and optical coherence tomography (OCT), the study demonstrated that evolocumab-treated patients—despite being on background high-intensity statins—achieved significantly lower LDL-C levels and a greater proportion reached optimal targets. The primary endpoint, reflecting improvements in fibrous cap thickness in the most vulnerable segments, was substantially better in the evolocumab group. Reductions in lipid content and inflammation (as evidenced by decreased lipid arcs and macrophage presence) were also more pronounced, particularly in initially lipid-rich plaques. Evolocumab also led to greater overall plaque regression than in the GLAGOV study (− 2.29% vs. − 0.95%) [35]. The PACMAN-AMI trial (NCT03067844) further evaluated alirocumab (150 mg biweekly) versus placebo over 12 months in post-MI patients using multimodal imaging. After one year, the alirocumab group achieved a significantly lower mean LDL-C (24 mg/dL vs. 75 mg/dL). IVUS showed a more marked reduction in atheroma volume (− 2% vs. − 1%), and OCT revealed a greater increase in fibrous cap thickness (+ 63 μm vs. + 32 μm) and a larger reduction in lipid core burden (− 80% vs. − 38%) and macrophage angle (− 26.0° vs. − 16.0°) [36]. Additional details on these pivotal clinical trials are summarized in Table 1.

**Table 1:** Main studies about PCSK9 inhibitors

Trial, NCT	No. of patients	Intervention/Treatment	Study type	Phase
FOURIER NCT01764633	27,564	Evolocumab Placebo	Interventional	III
ODYSSEY NCT01663402	18,924	Alirocumab Placebo LMT	Interventional	III
GLAGOV NCT01813422	970	Evolocumab Placebo	Interventional	III
HUYGENS NCT03570697	164	Evolocumab Placebo Statin therapy	Interventional	III
PACMAN-AMI NCT03067844	294	Alirocumab	Interventional	III
GAUSS NCT01763905	307	Evolocumab Placebo to Evolocumab Ezetimibe Placebo to Ezetimibe	Interventional	III
EVOPACS NCT03287609	308	Evolocumab Placebo	Interventional	III
ARCHITECT NCT05465278	104	Alirocumab	Interventional	IV
ODYSSEY ALTERNATIVE NCT01709513	314	Atorvastatin Ezetimibe Alirocumab Placebo	Interventional	III

*LMT* Lipid-modifying therapy

Based on current evidence, anti-PCSK9 monoclonal antibodies represent a viable therapeutic option for individuals who are unable to tolerate statins. In the GAUSS study (NCT01763905), evolocumab demonstrated significant LDL-C reductions ranging from 41 to 63% in statin-intolerant patients, without an associated increase in adverse events [37]. These results were further corroborated by the ODYSSEY ALTERNATIVE trial (NCT01709513), which compared alirocumab to both ezetimibe and atorvastatin. Alirocumab led to LDL-C reductions nearly threefold greater than those achieved with ezetimibe (approximately 45% vs. 15%) and was associated with a lower incidence of muscle-related side effects compared to atorvastatin [38]. Beyond monoclonal antibodies, PCSK9 inhibition can also be achieved using inclisiran, a synthetic small interfering RNA (siRNA) designed to target and degrade PCSK9 mRNA, thereby suppressing hepatic PCSK9 production [39, 40]. Early-phase clinical trials have shown that inclisiran lowers circulating PCSK9 levels by approximately 70% and reduces LDL-C levels by about 40% relative to placebo [41]. Unlike monoclonal antibodies, which require dosing every 2 to 4 weeks, inclisiran maintains efficacy with biannual administration. The ongoing ORION-4 trial is evaluating the long-term cardiovascular effects of inclisiran in approximately 15,000 individuals with established ASCVD, randomly assigned to receive either inclisiran or placebo (NCT03705234) [42]. Current US and European guidelines recommend PCSK9 inhibitors as either adjunctive or alternative therapy for patients who fail to achieve LDL-C targets with statins and ezetimibe. When used in conjunction with statins, PCSK9 inhibitors offer additional LDL-C reductions of 43–64% [43]. Despite their potent lipid-lowering and cardiovascular protective effects, widespread adoption may be limited by their high cost and the need for subcutaneous administration [44, 45]. In line with the 2019 ESC/EAS guidelines, the anticipated clinical benefit of lipid-lowering interventions can be estimated for individual patients based on baseline LDL-C levels, estimated ASCVD risk, and treatment intensity. On average, moderate-intensity statin therapy yields an LDL-C reduction of approximately 30%, while high-intensity statin therapy achieves around 50%. Adding ezetimibe can increase this reduction to about 65%. The addition of a PCSK9 inhibitor to high-intensity statin therapy can lower LDL-C levels by up to 75%, and when combined with ezetimibe, reductions may approach 85%. A detailed summary of estimated LDL-C reductions associated with various lipid-lowering strategies is provided in Table 2 [46].

**Table 2:** Reduction of LDL levels [46]

Lipid-lowering agents	Average reduction in LDL levels
LIS	< 30%
MIS	< 50%
HIS	≥ 50%
HIS plus ezetimibe	65%
PCSK9 inhibitors	60%
HIS plus PCSK9 inhibitors	75%
HIS plus ezetimibe plus PCSK9 inhibitors	85%

*LDL* Low-density lipoprotein, *LLD* Lipid-lowering drugs, *LIS* Low-intensity statins, *MIS* Moderate-intensity statins, *HIS* High-intensity statins

### Bempedoic acid

Bempedoic acid is a novel lipid-lowering agent that exerts its effects through dual mechanisms: inhibition of ATP-citrate lyase (ACLY) and upregulation of low-density lipoprotein (LDL) receptor expression. ACLY is a cytosolic enzyme that catalyzes the conversion of citrate to acetyl-CoA, a precursor for cholesterol and fatty acid synthesis [47, 48]. By inhibiting ACLY, bempedoic acid reduces hepatic cholesterol synthesis, leading to decreased intracellular cholesterol levels. This reduction triggers a compensatory upregulation of LDL receptors on hepatocyte surfaces, enhancing the clearance of circulating LDL cholesterol (LDL-C). Clinical studies have demonstrated that bempedoic acid effectively lowers LDL-C levels, both as monotherapy and in combination with other lipid-lowering therapies, particularly in patients with statin intolerance. Beyond its lipid-lowering capabilities, bempedoic acid has shown potential benefits in metabolic disorders. In animal models, treatment with bempedoic acid resulted in significant reductions in hepatic triglyceride accumulation and improvements in markers of liver inflammation and fibrosis, suggesting its utility in conditions like non-alcoholic steatohepatitis (NASH). Furthermore, recent research indicates that the beneficial effects of bempedoic acid on hepatic steatosis may not be solely attributable to ACLY inhibition. Studies have shown that bempedoic acid can ameliorate liver fat accumulation even in the absence of hepatic ACLY, implying the involvement of additional pathways in its mechanism of action. In summary, bempedoic acid lowers LDL-C levels by inhibiting ACLY and increasing LDL receptor expression. Its additional effects on hepatic lipid metabolism and inflammation highlight its potential as a multifaceted agent in the management of hypercholesterolemia and associated metabolic disorders [49, 50]. Bempedoic acid exhibits high oral bioavailability, attributed to its small molecular size and efficient intestinal absorption [51]. Notably, its cholesterol-lowering action is selectively confined to tissues capable of converting the prodrug into its active form. This activation is mediated by very-long-chain acyl-CoA synthetase-1 (ACSVL1), an enzyme abundantly expressed in the liver but minimally present in skeletal muscle and kidneys. The absence of ACSVL1 in skeletal muscle likely explains the low incidence of myotoxicity with bempedoic acid, a side effect seen in up to 30% of statin-treated patients [51, 52]. Pharmacodynamic studies have demonstrated that bempedoic acid, when combined with a lower-dose statin, can achieve LDL-C reductions comparable to those of higher-dose statins, offering a strategy to minimize statin-associated adverse effects in patients requiring intensive lipid-lowering therapy [53]. Bempedoic acid has demonstrated efficacy both as monotherapy and in combination with ezetimibe or statins. It serves as an effective adjunct in patients who do not reach LDL-C targets with statins alone and provides an alternative for statin-intolerant individuals. In several phase II studies, bempedoic acid was administered at daily doses ranging from 40 to 240 mg, either alone or in combination with other lipid-lowering agents [54, 55]. One particularly notable trial evaluated the combination of bempedoic acid (180 mg) with ezetimibe (10 mg) and atorvastatin (20 mg), achieving a remarkable placebo-corrected 60.5% reduction in LDL-C levels within 6 weeks. In this study, 90% of participants reached target LDL-C levels (< 70 mg/dL), and 95% experienced reductions of ≥ 50% [56, 57]. The clinical development of bempedoic acid included four pivotal phase III trials: CLEAR Harmony (NCT02666664) and CLEAR Wisdom (NCT02991118) enrolled patients with ASCVD or heterozygous familial hypercholesterolemia receiving maximally tolerated statin therapy [58, 59]. CLEAR Serenity (NCT02988115) and CLEAR Tranquility (NCT03001076) targeted statin-intolerant populations [60, 61]. Across all four trials, participants were randomized to receive either bempedoic acid or placebo. In CLEAR Tranquility, all participants received background ezetimibe therapy, with 30% also receiving low-intensity statins. In CLEAR Serenity, < 10% of participants received low-dose statins due to prior intolerance. In contrast, approximately 90% of participants in CLEAR Harmony and CLEAR Wisdom were on moderate- or high-intensity statins; 29–30% had diabetes, and 80% had hypertension. Despite differences in patient populations and background therapy, bempedoic acid consistently achieved significant LDL-C reductions at 12 weeks compared to placebo (*p* < 0.001 in all trials). Among patients on maximal lipid-lowering therapy, LDL-C reductions ranged from 17.4 to 18.1%, whereas in statin-intolerant cohorts, reductions ranged from 22 to 29%. These effects were sustained for up to 53 weeks and were paralleled by favorable changes in HDL-C, total cholesterol, and apoB levels. Collectively, these findings support the use of bempedoic acid as an effective lipid-lowering strategy for patients with ASCVD and/or familial hypercholesterolemia, either in combination with other agents or as monotherapy in statin-intolerant individuals. Importantly, all four phase III trials reported significant reductions in high-sensitivity C-reactive protein (hsCRP), a marker of systemic inflammation and predictor of cardiovascular events, suggesting potential anti-inflammatory benefits [62]. Bempedoic acid also demonstrated a favorable safety profile. Across trials, incidences of nasopharyngitis, urinary tract infections, and arthralgia were lower compared to placebo. When used alone or in combination with ezetimibe or statins (including fixed-dose combinations), it did not significantly increase muscle-related adverse effects such as myalgia or weakness. In statin-intolerant patients, it did not exacerbate preexisting muscle symptoms, reinforcing its suitability in this population. The cardiovascular efficacy of bempedoic acid was definitively assessed in the CLEAR Outcomes trial (NCT02988115), which enrolled 13,970 patients at high cardiovascular risk who were unable or unwilling to take recommended statin doses. Participants were randomized to receive either bempedoic acid (*n* = 6,992) or placebo (*n* = 6,978) and were followed for a median of 40.6 months. Bempedoic acid reduced the risk of major cardiovascular events—including cardiovascular death, non-fatal myocardial infarction, non-fatal stroke, and coronary revascularization—by 13% relative to placebo (11.7% vs. 13.3%). It also reduced secondary cardiovascular events by 15% (8.2% vs. 9.5%), with a particularly notable 23% reduction in myocardial infarction (3.7% vs. 4.8%) and a 19% reduction in coronary revascularization (6.2% vs. 7.6%) [63]. These findings strongly support bempedoic acid as an effective and well-tolerated agent for both LDL-C reduction and cardiovascular risk mitigation, especially in patients who are statin-intolerant or require combination therapy (Table 3).

**Table 3:** Phase III CLEAR trial series

Study, NCT, Ref	No. of patients	Baseline LDC (Bempedoic acid Vs Placebo)	% change of LDL	Adverse effects (Bempedoic acid)
CLEAR Harmony NCT02666664 58	2230	103.6 ± 29.1 mg/dL (BA) 102.3 ± 30.0 mg/dL (PL)	Mean difference: - Week 12: -18.1% - Week 52: -13.6%	Nasopharyngitis; Muscle spasm; Gout
CLEAR Wisdom NCT02991118 59	779	119.4 mg/dL (BA) 122.4 mg/dL (PL)	PL-corrected mean difference: - Week 12: -17.4% - Week 24: -14.8%	Urinary tract infection; Elevated uric acid; Muscle spasm
CLEAR Serenity NCT02988115 60	345	158.5 ± 40.4 mg/dL (BA) 155.6 ± 38.8 mg/dL (PL)	PL-corrected percent change: - Week 12: -21.4% - Week 24: -18.9%	Arthralgia; Pain in extremity; Gout
CLEAR Tranquility NCT03001076 61	269	129.8 mg/dL (BA) 123.0 mg/dL (PL)	Percentage change adjusted for placebo: After 12 weeks = -28.5%	Increased serum uric acid; Nausea; Sinusitis, Nasopharyngitis

*BA* bempedoic acid, *PL* placebo

### Other therapeutic developments

Hypercholesterolemia, particularly homozygous familial hypercholesterolemia (HoFH), presents significant therapeutic challenges due to markedly elevated low-density lipoprotein cholesterol (LDL-C) levels and increased cardiovascular risk. Recent advancements have introduced novel pharmacological agents targeting distinct pathways to manage this condition. Evinacumab, a monoclonal antibody inhibiting angiopoietin-like protein 3 (ANGPTL3), has demonstrated substantial LDL-C reduction in HoFH patients, independent of LDL receptor functionality. Lomitapide, an inhibitor of microsomal triglyceride transfer protein, effectively lowers LDL-C by impeding very-low-density lipoprotein assembly, offering benefits in both adult and pediatric HoFH populations. Mipomersen, an antisense oligonucleotide targeting apolipoprotein B-100 mRNA, reduces LDL-C levels but is associated with hepatic adverse effects, limiting its widespread use. Elevated lipoprotein(a) [Lp(a)] is an independent cardiovascular risk factor inadequately addressed by conventional therapies. Pelacarsen, an antisense oligonucleotide, significantly lowers Lp(a) concentrations and is under investigation for its potential to reduce cardiovascular events. Similarly, olpasiran, a small interfering RNA (siRNA) molecule, has shown prolonged Lp(a) reduction, maintaining decreased levels up to a year post-administration. Beyond pharmacotherapy, post-translational modifications (PTMs) such as glycosylation and phosphorylation play crucial roles in lipid metabolism regulation. Glycosylation affects lipoprotein structure and function, influencing atherogenicity. Phosphorylation modulates the activity of enzymes and transcription factors involved in lipid homeostasis, with implications for therapeutic targeting. Understanding these PTMs offers insights into novel intervention strategies for dyslipidemia and associated cardiovascular diseases [64–70].

## Conclusions

Despite substantial advances in reducing the incidence and mortality associated with atherosclerotic cardiovascular disease (ASCVD) in recent years across several European countries, ASCVD remains one of the leading causes of morbidity and mortality worldwide. The 2021 ESC Guidelines on cardiovascular disease prevention emphasize the paramount importance of achieving recommended LDL-C target levels, particularly in patients at high and very high cardiovascular risk [71]. However, reaching these targets remains a significant clinical challenge: At least 80% of high-risk patients require combination therapy to meet the new LDL-C goals [72]. While the combination of statins and ezetimibe has demonstrated efficacy in approximately 50% of patients, a considerable proportion still fails to achieve optimal LDL-C levels. This shortfall highlights the need for an additional therapeutic agent—ideally one that can be administered orally, or, when necessary, via injection [73]. Bempedoic acid presents a promising option in this context, offering a favorable tolerability profile, especially with respect to muscle-related adverse events—a key determinant of patient adherence. In addition to its LDL-C-lowering efficacy, bempedoic acid has been shown to reduce high-sensitivity C-reactive protein (hsCRP) levels, suggesting a potential role in addressing residual inflammatory cardiovascular risk [74]. This dual benefit may represent a meaningful advancement in the clinical and economic management of cardiovascular disease. From a cost-effectiveness perspective, the therapeutic landscape is currently polarized: While statins and ezetimibe are available at low annual costs (just over €100), PCSK9 inhibitors represent a substantially higher expenditure at approximately €6,500 per year. Within this framework, bempedoic acid offers a valuable alternative that could reduce dependence on costly PCSK9 inhibitors, yielding significant savings without compromising treatment outcomes. Such a shift could facilitate the reallocation of healthcare resources to other critical areas of care [75]. In conclusion, bempedoic acid enhances the therapeutic toolkit for managing high-risk patients and represents a strategic opportunity to optimize healthcare resource allocation. Its cost-effectiveness and clinical versatility position it as a potentially transformative agent in the contemporary management of cardiovascular risk.

## Data Availability

No datasets were generated or analysed during the current study.
